# Supplementary material for: GWAS on retinal vasculometry phenotypes
Source: PLoS Genet. 2023 Feb 9;19(2):e1010583. doi: 10.1371/journal.pgen.1010583 (PMC9910644; doi:10.1371/journal.pgen.1010583)
Supplement: S1 Acknowledgments — (DOCX) [file pgen.1010583.s001.docx]

**UK Biobank Eye and Vision Consortium**

Prof Naomi ALLEN

Prof Tariq ASLAM

Dr Denize ATAN

Prof Sarah BARMAN

Prof Jenny BARRETT

Prof Paul BISHOP

Prof Graeme BLACK

Dr Tasanee BRAITHWAITE

Dr Roxana CARARE

Prof Usha CHAKRAVARTHY

Dr Michelle CHAN

Dr Sharon CHUA

Dr Alexander DAY

Dr Parul DESAI

Prof Bal DHILLON

Prof Andrew DICK

Dr Alexander DONEY

Dr Cathy EGAN

Prof Sarah ENNIS

Prof Paul FOSTER

Dr Marcus FRUTTIGER

Dr John GALLACHER

Prof David (Ted) GARWAY-HEATH

Dr Jane GIBSON

Prof Jeremy GUGGENHEIM

Prof Chris HAMMOND

Prof Alison HARDCASTLE

Prof Simon HARDING

Dr Ruth HOGG

Dr Pirro HYSI

Prof Pearse KEANE

Prof Sir Peng Tee KHAW

Dr Anthony KHAWAJA

Mr Gerassimos LASCARATOS

Dr Thomas LITTLEJOHNS

Prof Andrew LOTERY

Prof Phil LUTHERT

Dr Tom MACGILLIVRAY

Dr Sarah MACKIE

Dr Bernadette MCGUINNESS

Dr Gareth MCKAY

Dr Martin MCKIBBIN

Prof Tony MOORE

Prof James MORGAN

Prof Richard ORAM

Dr Eoin O'SULLIVAN

Prof Chris OWEN

Dr Praveen PATEL

Dr Euan PATERSON

Dr Tunde PETO

Dr Axel PETZOLD

Dr Nikolas PONTIKOS

Prof Jugnoo RAHI

Prof Alicja RUDNICKA

Prof Naveed SATTAR

Dr Jay SELF

Dr Panagiotis SERGOUNIOTIS

Prof Sobha SIVAPRASAD

Prof David STEEL

Ms Irene STRATTON

Dr Nicholas STROUTHIDIS

Prof Cathie SUDLOW

Dr Zihan SUN

Dr Robyn TAPP

Dr Dhanes THOMAS

Prof Emanuele TRUCCO

Prof Adnan TUFAIL

Dr Ananth VISWANATHAN

Dr Veronique VITART

Dr Mike WEEDON

Dr Katie WILLIAMS

Prof Cathy WILLIAMS

Prof Jayne WOODSIDE

Dr Max YATES

Dr Jennifer YIP

Dr Yalin ZHENG
