# Supplementary material for: GWAS on retinal vasculometry phenotypes
Source: PLoS Genet. 2023 Feb 9;19(2):e1010583. doi: 10.1371/journal.pgen.1010583 (PMC9910644; doi:10.1371/journal.pgen.1010583)

**S1_Text: Supplementary Figures**

Contents

[**Fig A** 2](#_Toc124167806)

[**Fig B** 4](#_Toc124167807)

[**Fig C** 7](#_Toc124167808)

[**Fig D** 8](#_Toc124167809)

[**Fig E** 10](#_Toc124167810)

**Fig A. Manhattan plots depicting the genome-wide associations for arteriolar tortuosity (i), arteriolar width (ii), venular tortuosity (iii) and venular width (iv).** Each dot represents one polymorphism, the x-axes show the chromosomal location and the position within the respective chromosomes. The y-axes show the log_10_(p-value) from the respective analyses. Annotations of selected loci (the most significant) show the nearest gene at the locus where appropriate. The most significant associations with each trait are labelled as red dots. The dark dash line indicates the threshold for suggestive associations (P < 5×10^-8^).

**(i) AT**

**
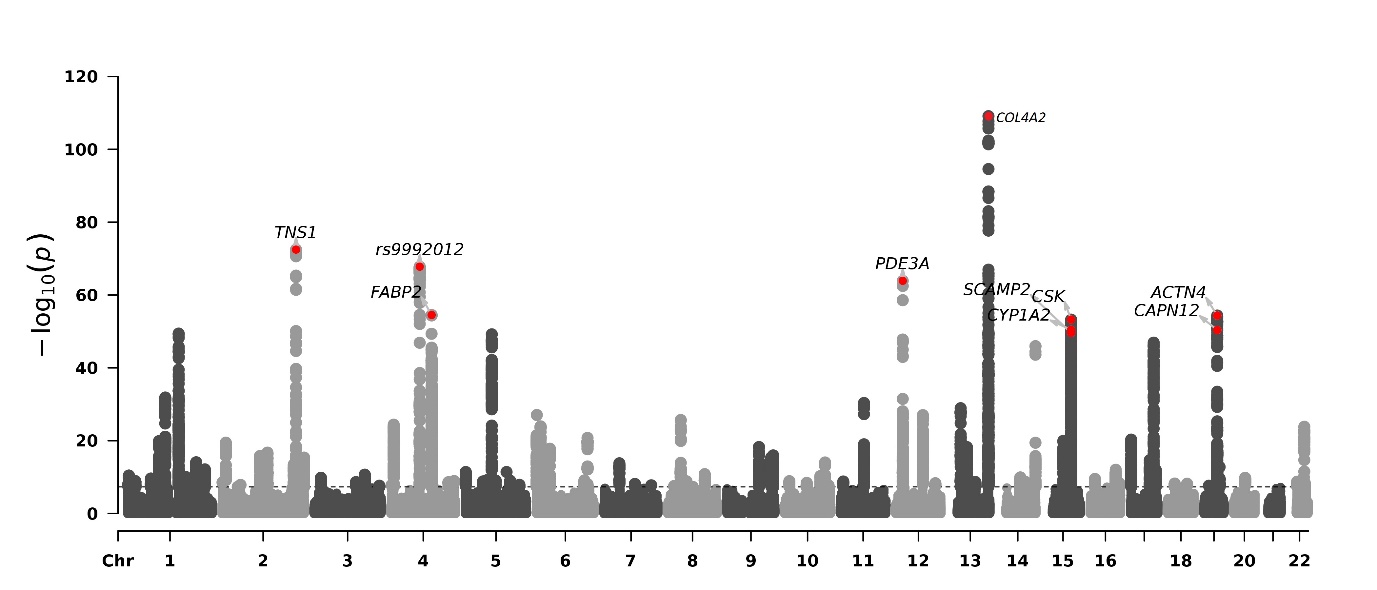
**

**(ii) AW**

**
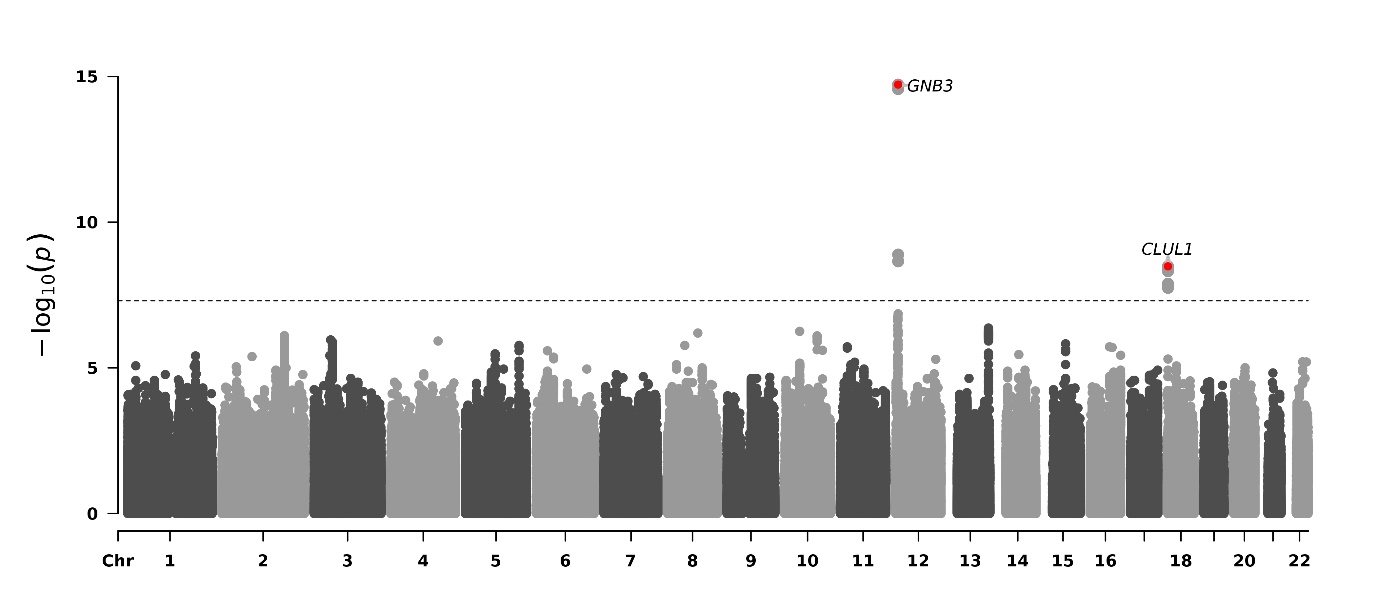
**

**(iii) VT**


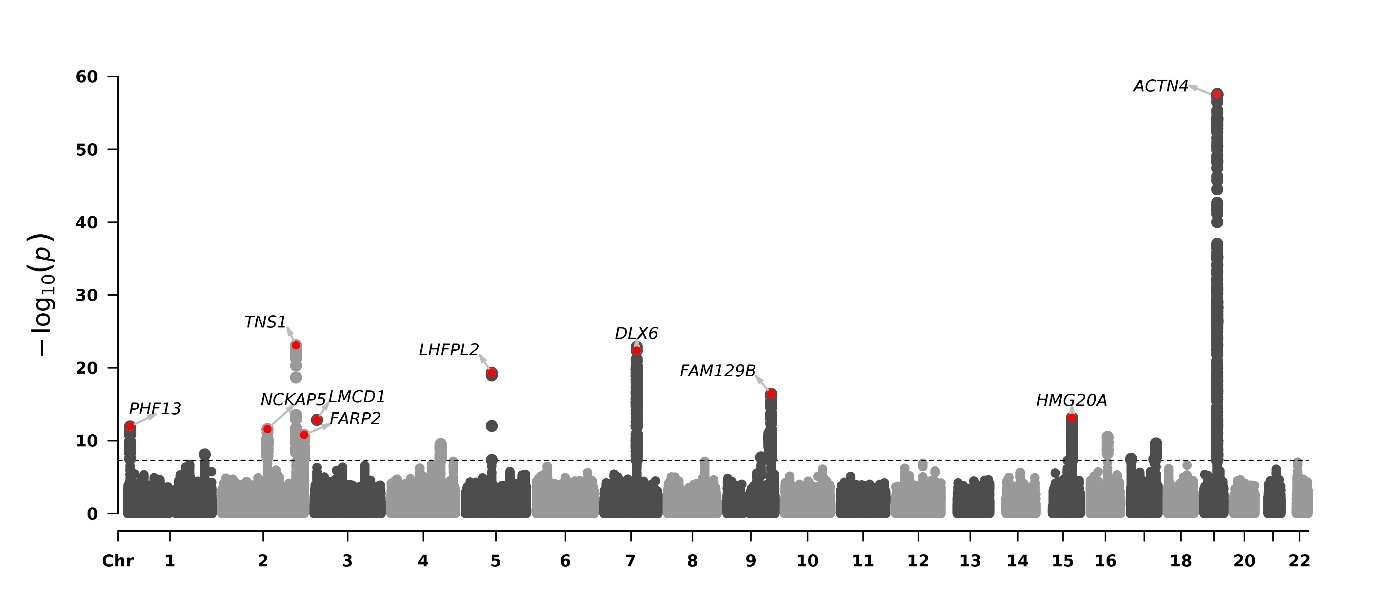


**(iv) VW**


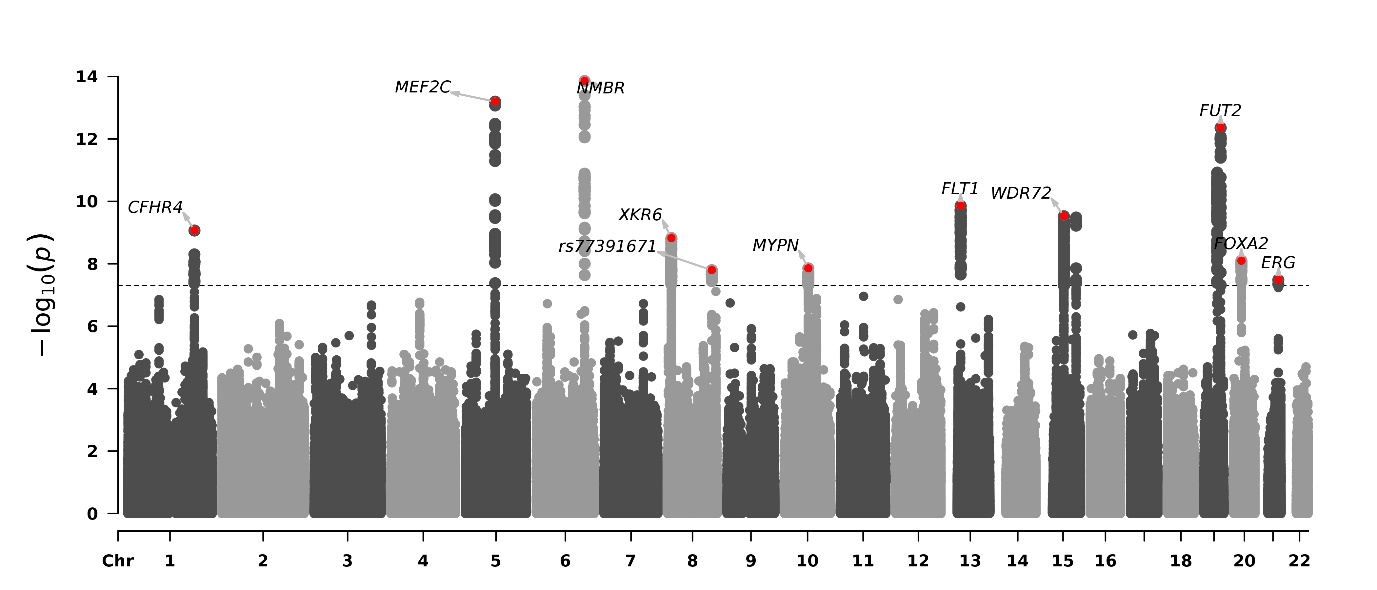


**Fig B. The relationship between effects sizes estimated in association with retinal vessel parameters in the UK Biobank (x-axes) and those estimated in the EPIC-Norfolk cohort (y-axes).** Each green dot represents one SNP and the projections on each axes correspond to the estimated effect sizes. The black solid lines denote the linear regression line and the grey shaded areas the 95% Confidence Intervals. Labels represent the nearest genes, as shown in the S1_Table C. Some of these labels may have been omitted for the sake of clarity. Comparisons for three different parameters are shown: (i) arteriolar tortuosity, (ii) venular tortuosity, (iii) venular width. The number of associations observed for the arterial width was too small to allow meaningful comparisons and are therefore omitted.

**(i)**


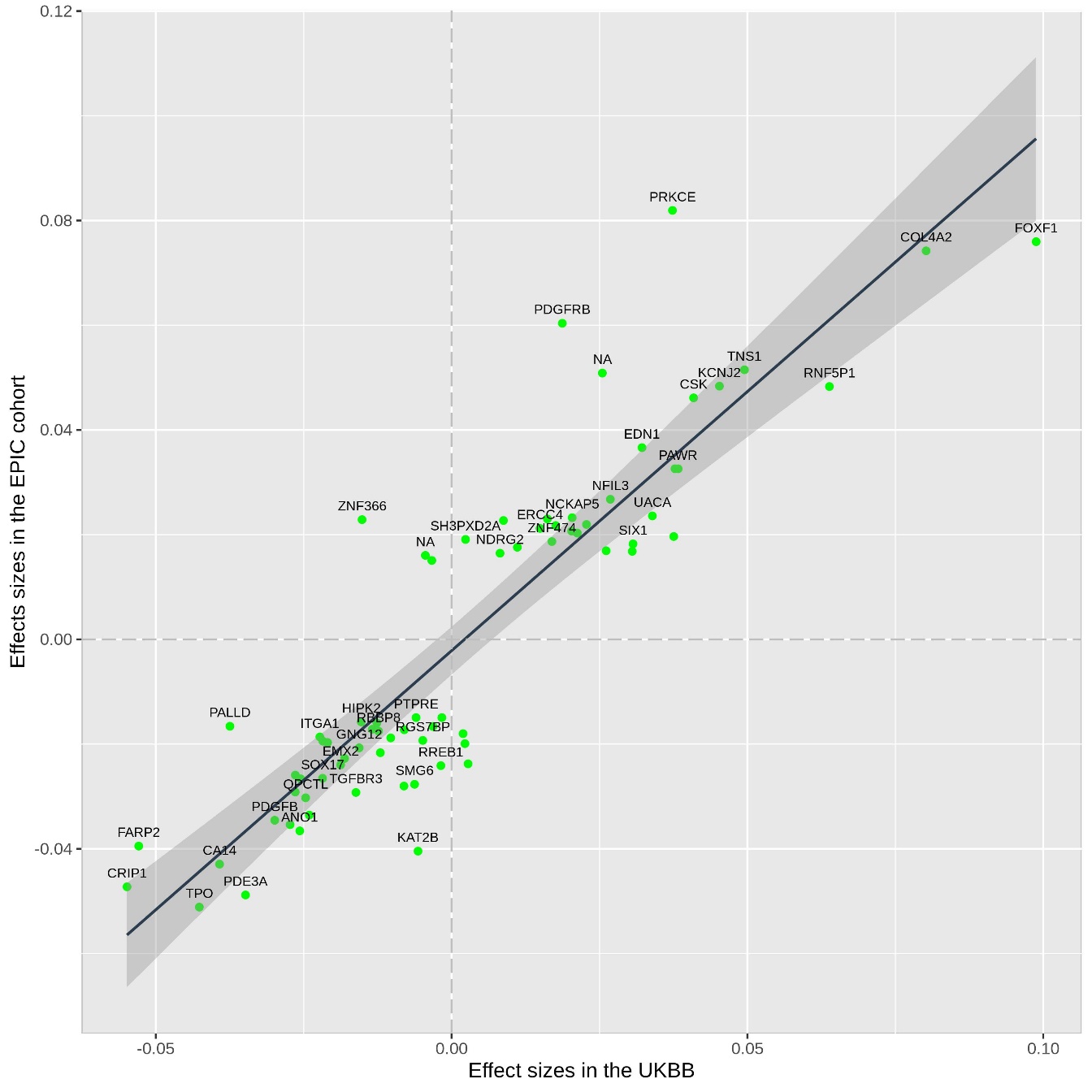


**(ii)**

***
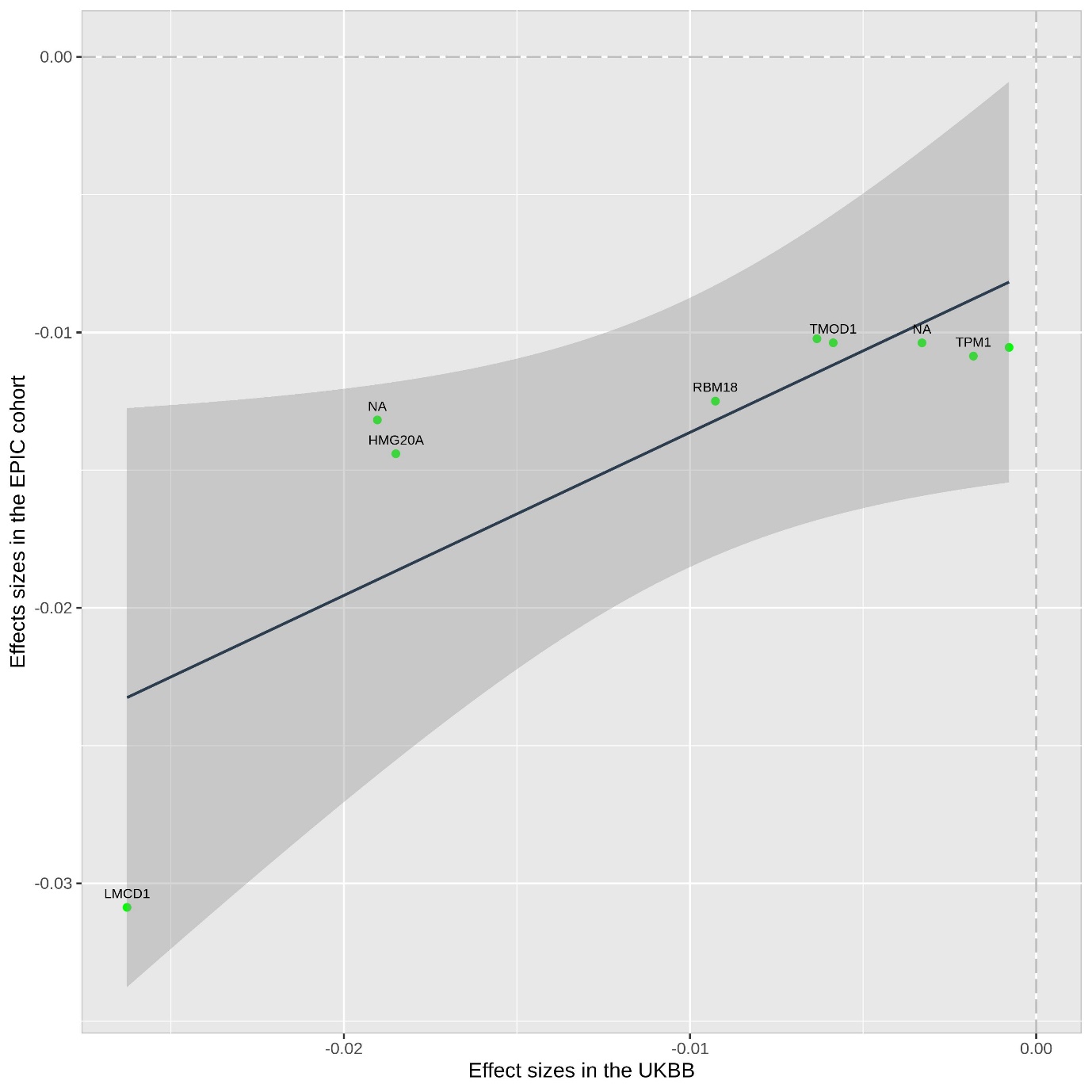
***

**(iii)**

***
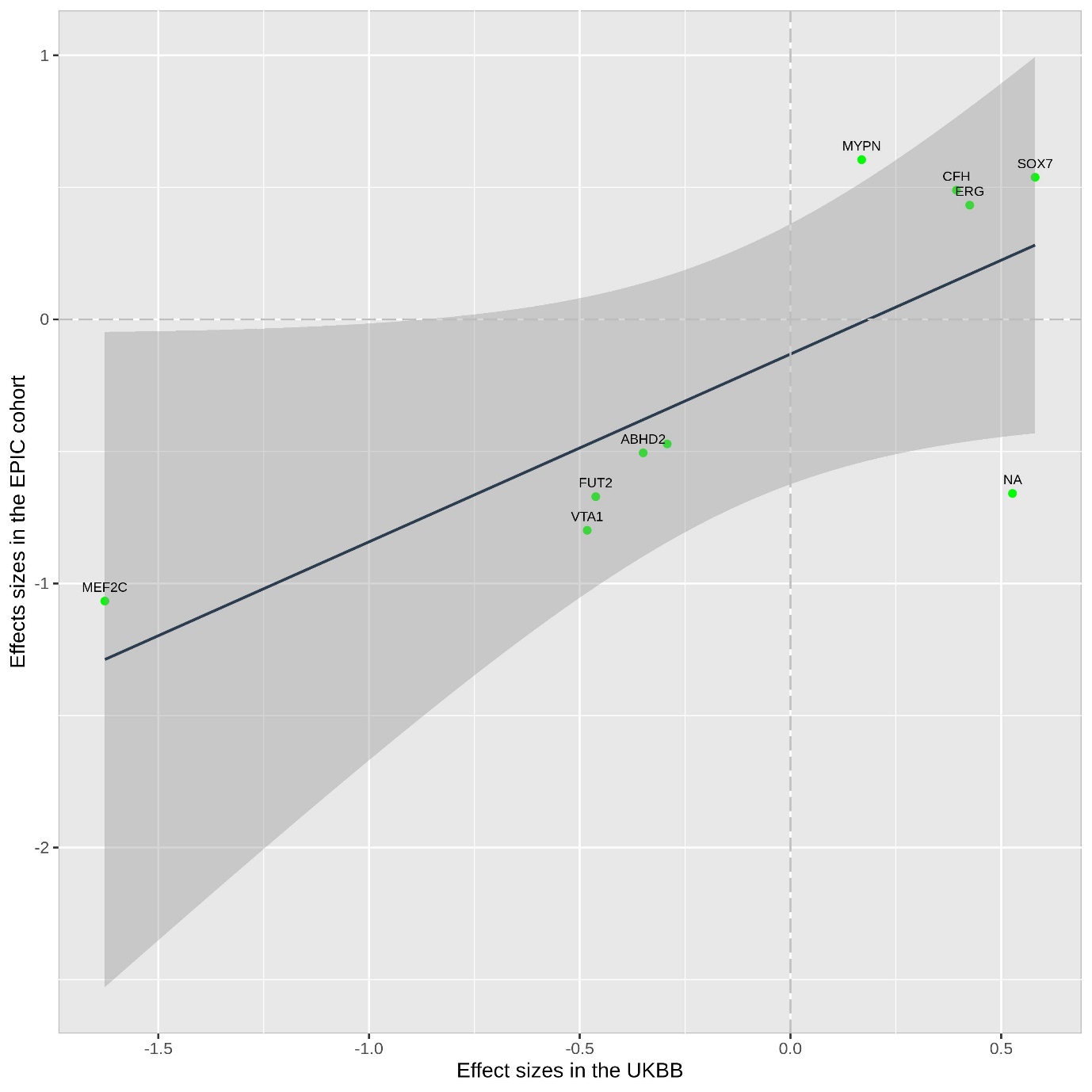
***

**Fig C. The relationship between effects sizes estimated in association with retinal vessel parameters in the UK Biobank (x-axes) and those estimated in the Africa/Asian ancestry (y-axes).** Each green dot represents one SNP and the projections on each axes correspond to the estimated effect sizes. The blue solid lines denote the linear regression line and the grey shaded areas the 95% Confidence Intervals. Labels represent the nearest genes, as shown in the S1_Table F. Some of these labels may have been omitted for the sake of clarity. Comparisons for arteriolar tortuosity are shown: (i) African vs. Europe, (ii) Asian vs. European.

**(i)*
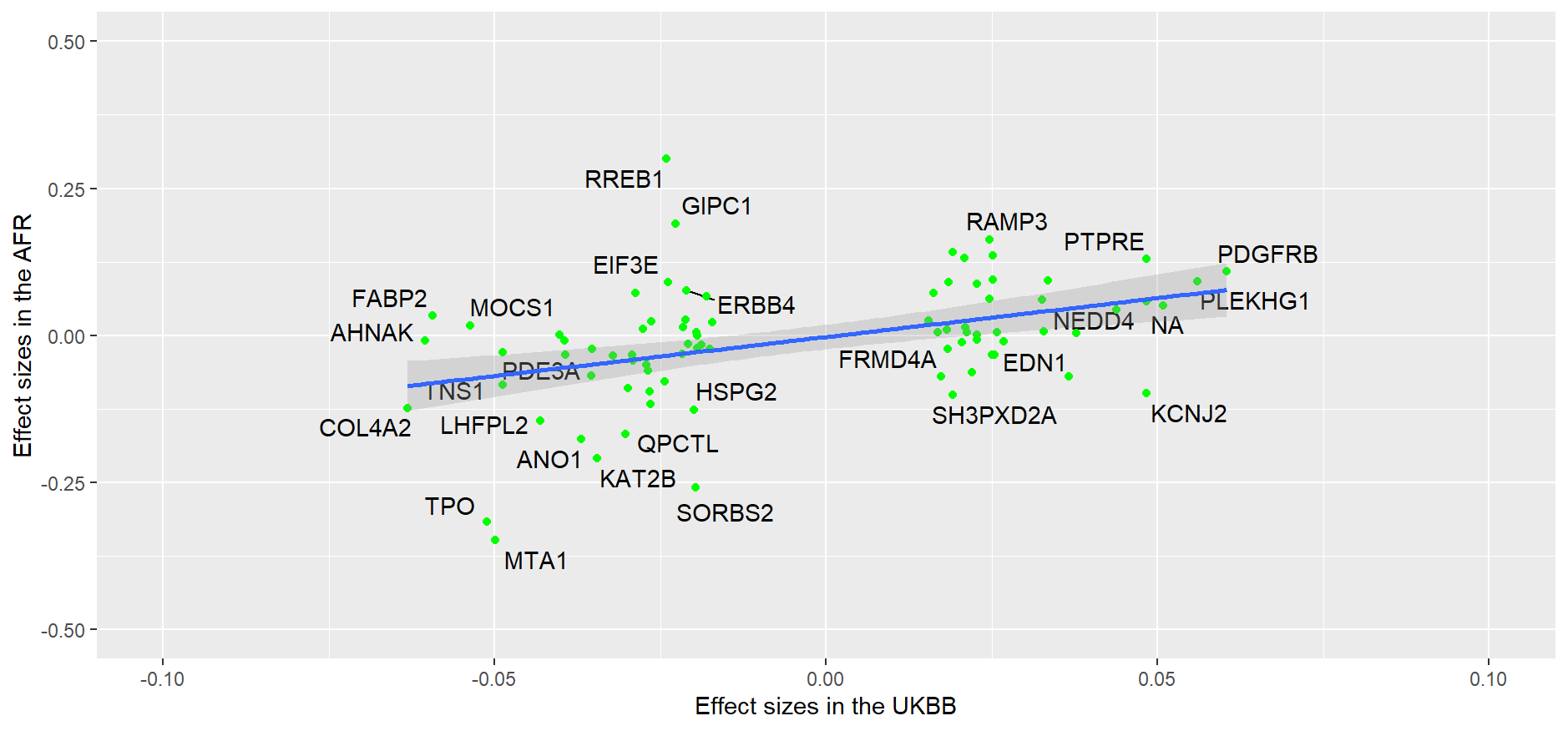
*(ii)*
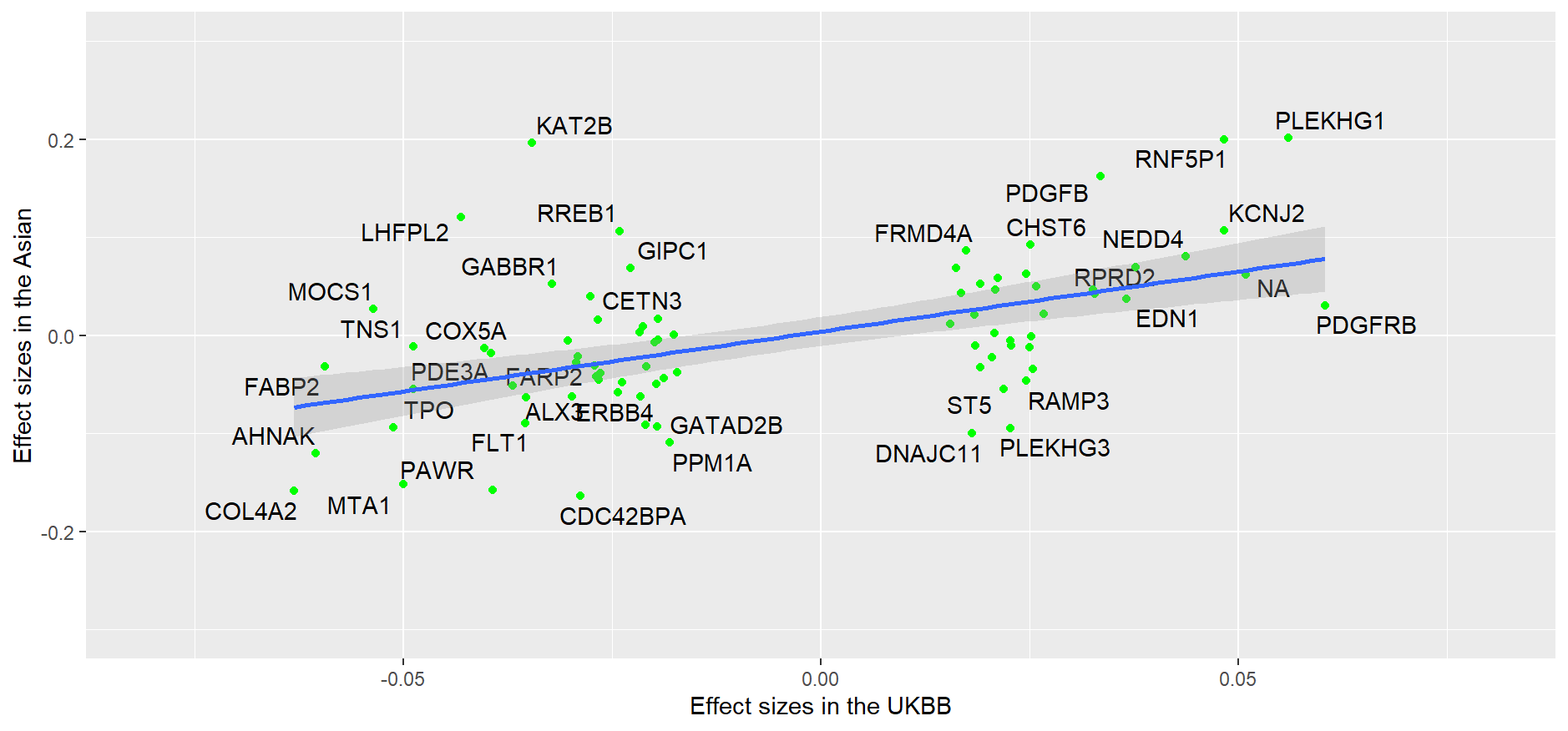
***

**Fig D. Tissue expression enrichment analysis results, for AT (i), AW (ii), VT (iii) and VW (iv).** The different tissues are grouped along the x-axes and share the same colour code shown in the legends. Each point represents one tissue; the y-axes show the statistical significance for the enrichment. The most significantly enriched tissues are labelled.

**(i)**


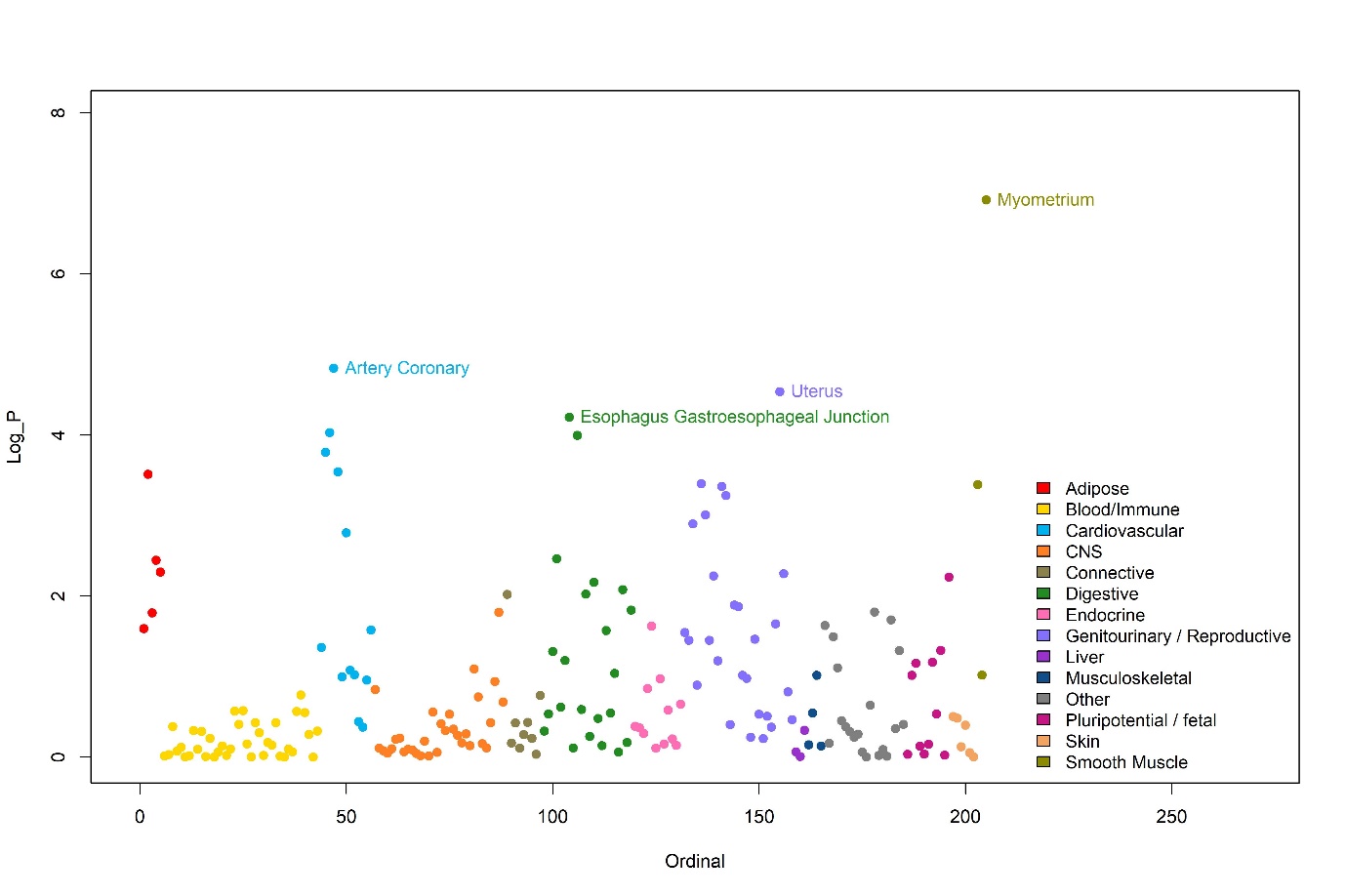
**(ii)**


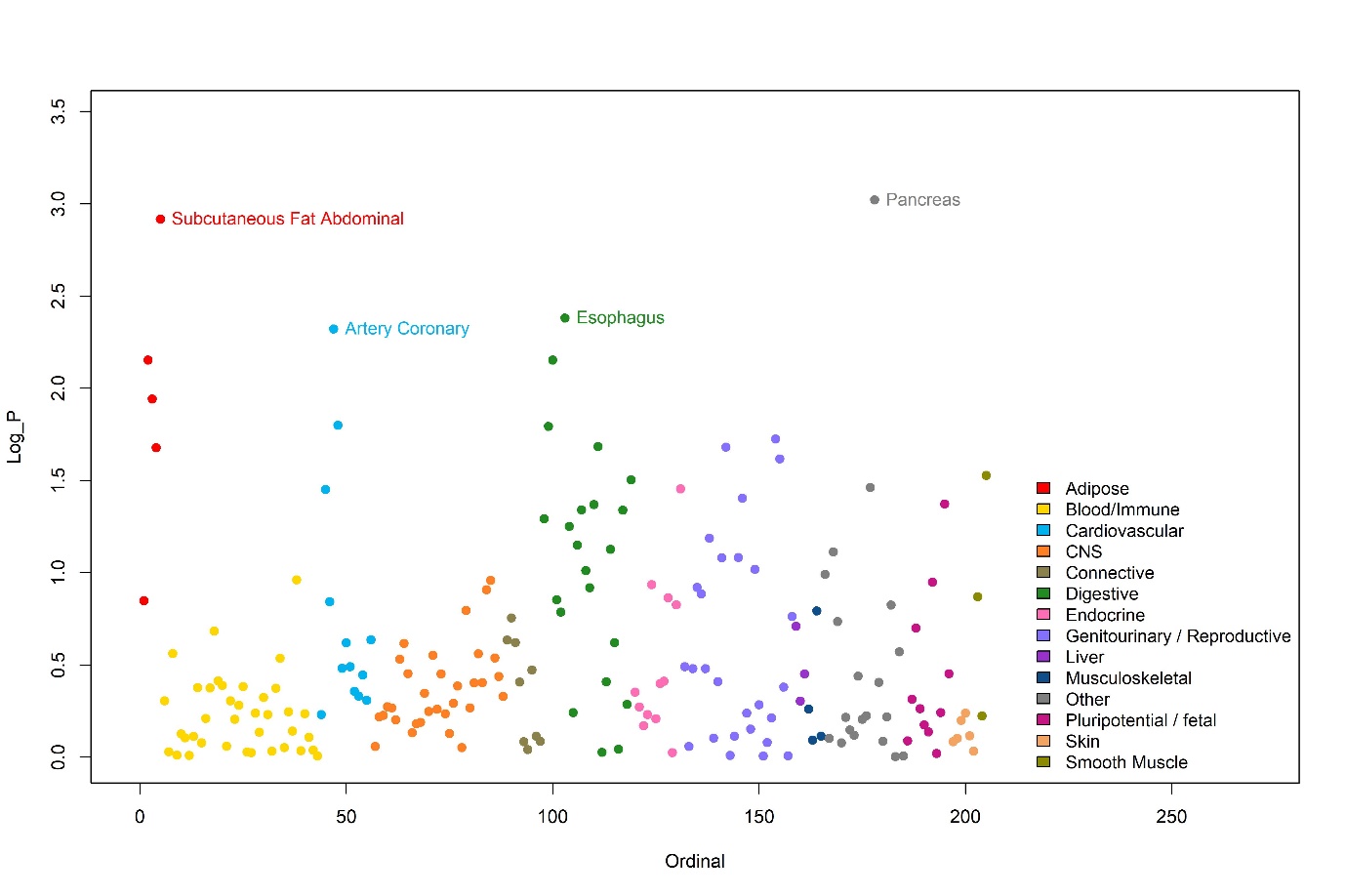


**(iii)**


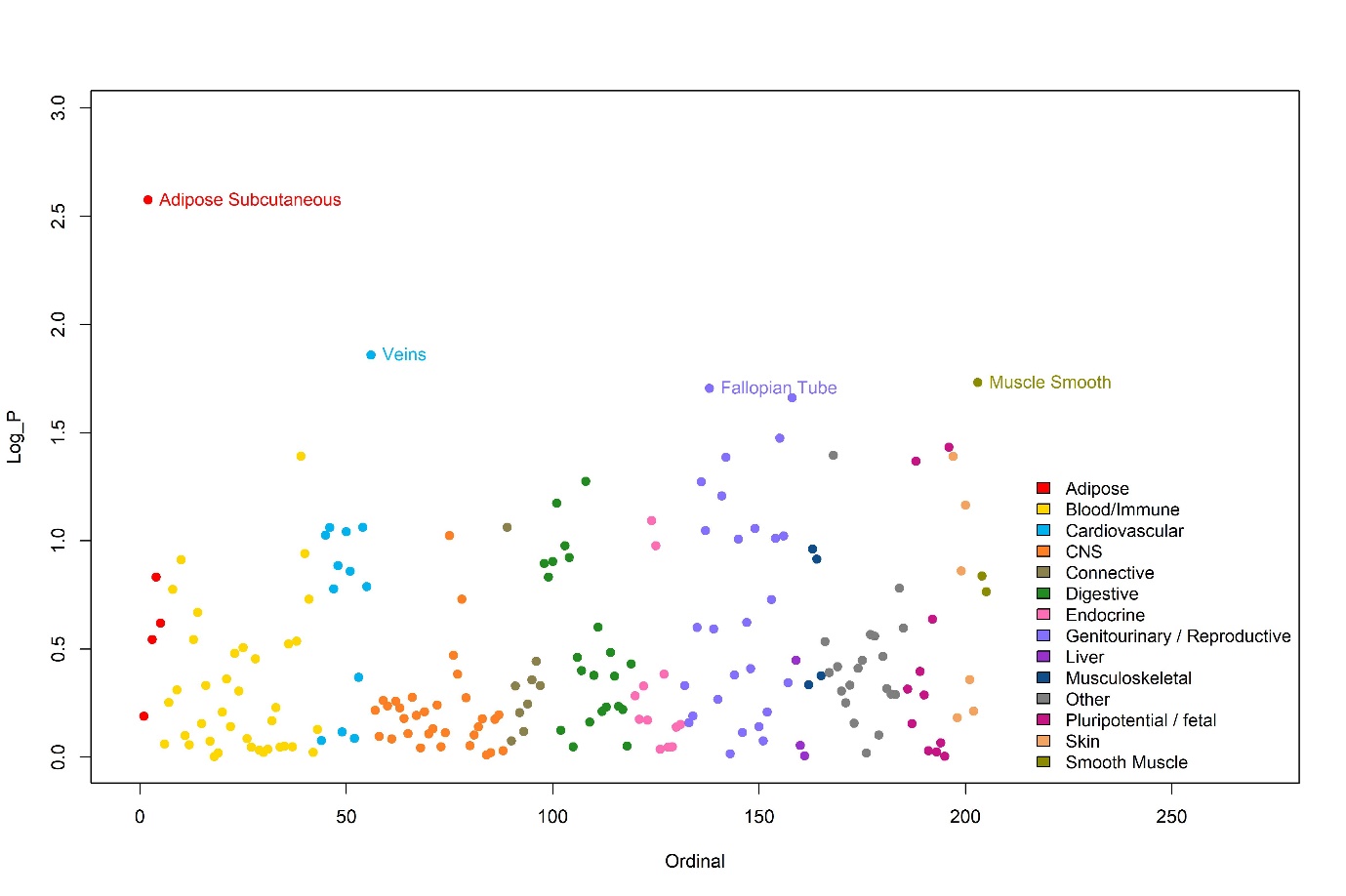


**(iv)**


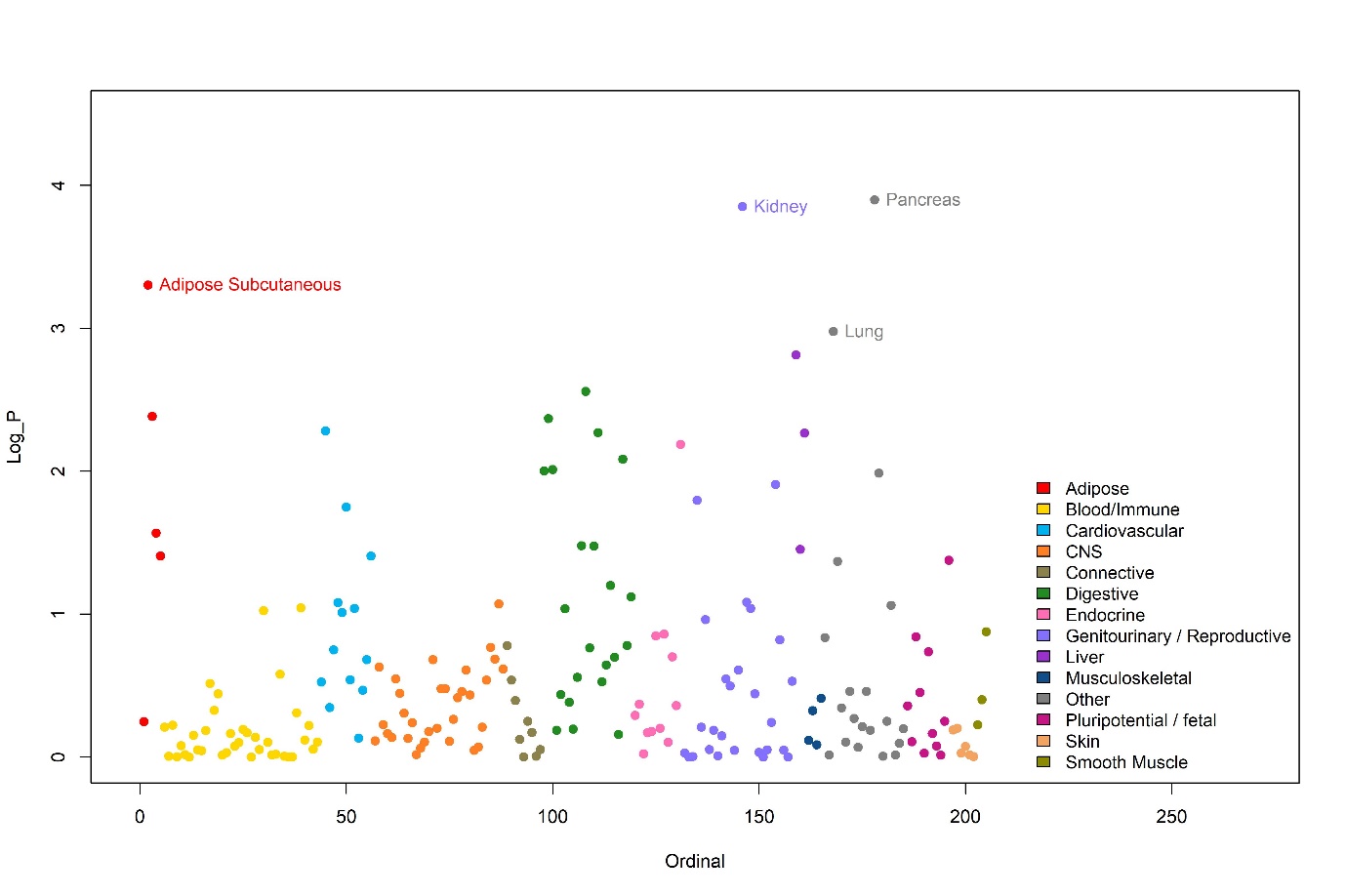


**Fig E. SMR Integration of results combined GWAS and eQTL for AT on Peripheral blood.** The innermost circle, middle circle and outer circle indicates the eQTL, GWAS and SMR results, respectively. Separated y-axis was shown in each circle.
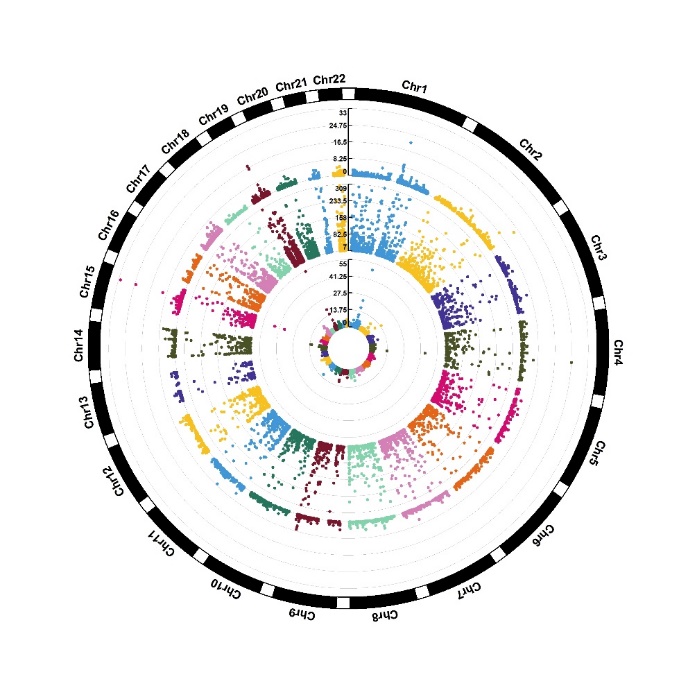

Supplement: S1 Text — Fig A. Manhattan plots depicting the genome-wide associations for arteriolar tortuosity (i), arteriolar width (ii), venular tortuosity (iii) and venular width (iv). Fig B. The relationship between effects sizes estimated in association with retinal vessel parameters in the UK Biobank (x-axes) and those estimated in the EPIC-Norfolk cohort (y-axes). Fig C. The relationship between effects sizes estimated in association with retinal vessel parameters in the UK Biobank (x-axes) and those estimated in the Africa/Asian ancestry (y-axes). Fig D. Tissue expression enrichment analysis results, for AT (i), AW (ii), VT (ii) and VW (iv). Fig E. SMR Integration of results combined GWAS and eQTL for AT on Peripheral blood. (DOCX) [file pgen.1010583.s002.docx]
