# Supplementary material for: GWAS on retinal vasculometry phenotypes
Source: PLoS Genet. 2023 Feb 9;19(2):e1010583. doi: 10.1371/journal.pgen.1010583 (PMC9910644; doi:10.1371/journal.pgen.1010583)
Supplement: Attachment — Submitted filename: PGENETICS-D-22-00757_Response to reviewers (141122).docx [file pgen.1010583.s004.docx]

**Reviewer's Responses to Questions**

**Comments to the Authors:**

**Reviewer #1: The manuscript by Jiang et al describes genome-wide analyses of retinal vascular parameters with several bioinformatic analyses designed to identify phenotypic overlap. The manuscript is largely well-written and fairly clear, however there are some elements which would benefit from additional clarification. Broadly speaking, it would add quite a bit to the manuscript to discuss the results in the context of gene regulation across your various tissues in addition to phenotypic causation.**

*We thank the Reviewer for their positive comments about our manuscript. In response to the request for further explanation, we have added text to clarify the commonality of genetic associations across different tissues, particularly those primarily involved in cardiac function. We have also reviewed and updated the text in relation to gene expression pathways, which potentially relate to the findings.*

**1. Is there overlap between EPIC-Norfolk and the UKB participants included in this study? Given the similar catchment areas it would be best to include a statement of independent samples.**

*There were 22 baseline centres in UK Biobank but only 6 eye centres, which were located in Croydon, Hounslow, Liverpool, Sheffield, Birmingham, and Swansea. Hence, there was no geographic overlap with Norwich. While we are unable to interrogate the data to formally ascertain overlap (due to the legal obligation to respect the anonymity of the participants and their data) we believe that this is very unlikely. We have added a statement to the ‘Methods’, ‘Statistical Methods’, ‘Replication GWAS - EPIC-Norfolk’ section (page 5, lines 159-162 of the revised article in track changes) to reassure over the geographic independence of the validation dataset (i.e., EPIC-Norfolk) in relation to the test data (UK Biobank).*

**2. Were the parameters similar for the LDSC and LD Hub analyses? Specifically LDSC recommends filtration to HapMap SNPs, and use of stringent info score thresholds, were identical thresholds applied in both approaches?**

*All the parameters in LDSC can be set in the LD Hub. Hence, the LD Hub results could always be validated on LDSC. We have added the following statement to the ‘Methods’ (page 5, lines 185-186):*

*“All the parameters used for the LDSC analyses were following the authors’ recommendations ^1^ for both the analyses using the LD Hub GWAS information and our own.”*

**3. Which version of GTEx was used for your S-PrediXcan analysis? 44 tissues would suggest use of v6 rather than the newer (and larger) v7 or v8, and if so ought to be stated. Also where are the results from these analyses? They're never discussed or presented.**

*We thank the reviewer for this observation. We have updated our results with the GTEx v8 dataset, where 49 tissues were applied. The results and corresponding study tissue-specific gene expressions shown in Supplemental Table 7 have been updated accordingly. We have amended the main text accordingly (page 6, lines 194-200) and made the results available in Supplemental Table 7.*

**4. The tissue enrichment methods should be described in bit more detail, specifically indications of the versions of databases used, etc. as the 200+ tissues interrogated are not listed in more detail. The results indicates GTEx tissues were used for enrichment, but that doesn't add up - there are not 200+ tissues available in GTEx, and myometrium isn't one of them. Please clarify.**

*We apologise for our lack of clarity. We used information from expression data derived from multiple tissues that include but are not limited to the GTEx data. To improve clarity, we have now amended the text (pages 5-6, lines 194-200) and the legend of Supplementary Figure 4.*

**5. I am puzzled by the selection of tibial artery rather than coronary artery as one of the tissues, given that coronary artery was a top signal in two of the four analyses.**

*We believe the reviewer is referring to our SMR analyses. Because of the importance of power and sample sizes, we opted to run analyses in the well powered tibial artery, for which information was available from N=584 individuals rather than the coronary artery (N=213). We have added the following sentence to the newly revised version of the manuscript (page 6, lines 207-209):*

*“To maximise power, we conducted these analyses in subcutaneous adipose (N = 581), tibial artery (N = 584, chosen a priori in preference to coronary artery due to great sample size), and a less specific dataset of less-tissue specific leukocytes for which larger sample sizes (N = 5,311) were available”.*

**6. The Manhattan plots show many significant signals, far more than are mentioned in the case of arteriolar width. If filters were applied, that needs to be reflected in what is plotted and described more thoroughly. If not, then the results are not described properly and there would be more than enough datapoints to enable comparison of effect sizes between EPIC-Norfolk and the UKB.**

*We thank the reviewer for bringing this to our attention and apologize for not applying the filter correctly. The previous figure included SNPs that were exceedingly rare and insufficiently well imputed. Apologizing for our previous mistakes we have filtered out all the unreliable results from that figure.*

**7. This isn't critical, but it would be nice to see figures analogous to Supplementary Figure 2 for the cross-ancestry analyses in addition to the supplementary table. This information should also be mentioned in the discussion paragraphs of particular gene results.**

*Two correlation plots of cross-ancestry results have now been added as new supplemental figures (Supplemental Figures 3 A and B).*

**8. Line 278: hereditability =** **heritability**

*Thanking the reviewer for spotting this unfortunate mistake, we have now corrected the spelling (page 8, line 298).*

**9. The cross-disease genetic correlation analyses seem highly unfocused. The numbers of phenotypes need to be clarified, preferably restricted to those with a scientific rationale ("Distance between home and job workplace"). Also ST9 lists all populations as UKBB, of which your discovery analyses is a subset, this is somewhat concerning.**

*We agree with the reviewer that our indiscriminate reporting of all results available from the LDSC portal, with some traits heavily influenced by socio-economic factors is confusing and we have removed them from the most recent version of that supplementary table. The LDSC regression does not assume samples independence and indeed genetic correlations can be calculated for traits studied in the same populations.*

**10. Previously identified results from reference 42 are mentioned in the second paragraph of the discussion, were these among the top hits in this manuscript, and/or were these SNPs evaluated systematically? Noting the replication of these loci would be useful.**

*The replication results between results from reference 42 (now reference number 16) ^2^ and our data are shown in Supplementary Table 12.*

**Reviewer #2:**

**The authors perform a large GWAS using four QUARTZ-derived fundus features as quantitative traits in 52,798 UK biobank individuals. While this is not the first GWAS on vascular features of the UK biobank fundus photos, this is an important topic of investigation given the link to systemic disease. The research methods in this paper are sound. I do think there needs to be substantial additional consideration to the analysis that I will discuss below. I have some general comments followed by more specific page/line comments:**

*We thank the Reviewer for recognising the importance of the work and our sound approach. We acknowledge the further points raised by the reviewer, which we feel we have been able to address (see below), resulting in an improved manuscript.*

**Image processing: I do not have experience with QUARTZ but it seems reasonable. However, I think that the description of imaging processing needs more detail (I looked in the supplemental but do not see any further detail)**. **The authors do not describe how they handle each eye – is one eye used? Both eyes? If both, how do they account for correlation? How do they handle significant differences between the two eyes and the possible confounding etiologies?**

*QUARTZ measures were summarised using mean width in microns and tortuosity (arbitrary units), weighted by the length of the vessel segment, for arterioles and venules separately for each image. Mean segment width and segment lengths were also used to calculate total arteriolar and venular total vessel areas in mm². The standard deviation (SD) of width measures along a vessel segment were obtained and averaged for each image weighted by segment length. Person level averages of all vessel indices were obtained by averaging measures between right and left eyes to give vessel indices. As we are largely examining systemic traits, we do not believe that we need to distinguish between eyes within an individual, as systemic influences will affect both eyes and not be localised.*

*We have now added further text to clarify use of person-levels means to the ‘Phenotyping and retinal imaging’ subheading in the ‘Methods’ section (page 3, lines 115-117).*

**How do the authors correct for image quality? This will degrade with heritable things like corneal disease and cataract, and I assume degradation of the image will systematically affect the 4 measurements.**

*Our automated image quality algorithm ^3^ showed that 77% (n/N=67777/88052) of UK Biobank participants and 80% (n/N=5957/7411) EPIC-Norfolk study participants had retinal images of sufficient quality for inclusion. The high level of image quality is because useful information can still be extracted from well segmented sections of the vasculature, even if this only represents a portion of the vascular tree, i.e., the entire image is not needed. Hence, we believe corneal disease and cataract are unlikely to have appreciably impacted on retinal vasculometry measures included in the analyses. The observed reasons for exclusion were multiple and included incorrect camera exposure, focal plane error, small pupils, eye lashes/blinking, and head/eye movement, in addition to lens artefacts, media opacity (related to corneal disease and cataract, which the reviewer appropriately raises).*

*We have added new text to the Discussion acknowledging the potential impact of corneal disease and cataract on retinal image quality and the consequences they may have on the heritability estimation of these traits (page 11, lines 423-426).*

**How about correcting for retinal pathology in general? Are these all patients without a diagnosis of certain major categories of ocular disease? For example: someone with a history of retinal laser (for diabetes, a retinal tear, a retinal detachment, etc) will have different vasculometry measurements due to the ablation of the retina in the area of laser. Traits leading to the need for laser are heritable.**

*We specifically excluded all pre-existing retinal pathologies in our analyses. At the time of writing, no data on clinically graded disease related retinal features have been deposited on the UK Biobank showcase as yet (see* [*https://biobank.ndph.ox.ac.uk/showcase/*](https://biobank.ndph.ox.ac.uk/showcase/)*). Self-reported ocular disease was not used due to concerns over validity and underreporting. While linkages to other relevant electronic medical record derived data would be possible (including primary care and hospital admission data), this is an incomplete and ongoing process.*

**The GWAS itself: the methods are sound and use well-established analysis pipelines. The authors correct for age, sex, spherical equivalent and the first ten principal components. My main concerns revolve around confounders and clear delineation about findings here that imply a novel pathway as separate from one that is already well understood by ophthalmologists. To be specific: Smoking status is not controlled for in this paper – it should be added as a covariate, as it has definite secondary effects on the vasculature throughout the body.**

*There may be a theoretical advantage in covariate-adjustment in that it may reduce the residual variance of the outcome and thereby increases power to detect covariate-independent genetic associations. However, in many circumstances adjustment for non-biological covariates (such as smoking) may be even harmful due to information missingness that reduce the sample size and further erode power due to recall and reporting bias. However, to respond constructively address the reviewer’s concerns, we have run models with adjustment for current smoking status, which generally appears to have a minimal effect. We have added a sentence to indicate that adding current smoking status as a covariate made very little difference and added a comparison between unadjusted and adjusted analyses as a new Supplementary Table 13.*

**Retinal vein occlusion (or impending vein occlusion), one of the more common secondary reasons for vascular tortuosity, is not mentioned in this paper. A sign of this would be a difference in tortuosity between the two eyes (in the case of CRVO and assuming it wasn’t bilateral) or a sectoral change within the eye (BRVO). Can the authors account for this in any way (there are likely several ways of doing this)?**

*Clinically graded retinal features indicative of occlusion are yet to be made available in UK Biobank. However, the prevalence of self-reported central retinal vein occlusion in the entire cohort is low (0.5%), and numbers will be very small among the subgroup with retinal images. We now have added text to the Discussion (page 10, line 422) to acknowledge CRVO as a cause of retinal vessel tortuosity and our inability to explicitly examine this in the current study as a potential limitation.*

**Systemic hypertension, cardiovascular disease, diabetes and (to a certain degree) hyperlipidemia are other well known clinical associations with vascular tortuosity on exam. In a way, it is not novel to find SNPs that co-associate with VT and these traits. Yet, there is no analysis here that controls for this. One could argue that controlling for these traits is not necessarily desired, because they are in the causal pathway, but it would be interesting to have a secondary analysis that controls for blood pressure and diabetes (for example) and reports SNPs that associate independent of this confounder that is already understood as associated with vascular tortuosity.**

*We agree with the reviewer that many conditions and phenotypes could directly or indirectly affect our phenotypes. However, here we aim to identify all genetic variants that affect retinal vasculature and that explain most of the phenotypic variance, regardless of the mechanisms through which they exert their influence. Regressing out the effect of intermediary phenotypes would contribute to the problem of “missing heritability” and could actually lead to biased effect estimates that would make derivative analyses such as Mendelian randomisation virtually impossible. This would decrease the value of our findings to the broader community****.***

**It is standard to correct for age-squared to model its effect and account more accurately for any non-linearity.**

*Variables such as age that are not associated with the independent variables (i.e., genotypes), may cause significant dispersion, but not bias. However, in our newly revised manuscript, we report a comparison of results before and after adjustment for the covariates suggested by the reviewer. As the adjusted results are fully in line with the unadjusted ones, we continue to refer to the unadjusted findings in the text of our manuscript.*

**Uncontrolled diabetes and hypertension (amongst other things) can lead to vascular narrowing and pruning and eventual non-perfusion of the peripheral retina. I assume the time with respect to disease onset will have an effect on the vasculometry measurements. Can the authors discuss how they handled this (or why they decided not to control for it)?**

*We believe we have addressed this issue in previous responses to reviewers’ concerns and suggestions.*

**I don’t believe genotyping array is added as a covariate and it likely should be if it varied in this large sample size**

*The UK BiLEVE Axiom array (used in about 10% of the UK Biobank sample) is part of the Biobank Axiom array used for the rest of the genotyping. Both are manufactured by Affymetrix (now part of ThermoFisher). The Axiom chip includes virtually all the UK BiLEVE Axiom SNPs. Correcting for possible differences in genotype calling between arrays was applied in the early UK Biobank GWAS. As more experience was gathered in the field, differences between the two chips were found to be illusive and most of the latest GWAS (many of which authored by us) have found it safe to ignore the trivial differences in one small batch of the UK Biobank.*

**Page 3 line 68: This description of the retinal vasculature is not correct. The** **long and short ciliary artery split off BEFORE penetrating the sclera. Together, the long posterior ciliary artery and the short posterior ciliary arteries feed the choroid (long and short), iris (long) and ciliary body (long).**

*We apologise for this incorrect anatomical description and have now corrected the sentence in the Introduction (page 3, lines 69-70).*

**Page 3 line 73: Retinal vein occlusion (or impending vein occlusion) should have been considered in this manuscript as a cause for venous tortuosity, especially if unilateral.**

*As previously mentioned, we have added a statement in the Discussion to outline Retinal Vein Occlusion as a potential cause of increased retinal vessel tortuosity.*

**Page 3 line 74: Cite Zekavat et al. Circulation 2022 as a paper that is already published using the UK biobank to study retinal vasculature. Please alter statements about novelty and explain the distinction between VT/VD and the traits in the Zekavat paper. Also cite this work:** [**https://www.medrxiv.org/content/10.1101/2020.06.25.20139725v6**](https://eur03.safelinks.protection.outlook.com/?url=https%3A%2F%2Fwww.medrxiv.org%2Fcontent%2F10.1101%2F2020.06.25.20139725v6&data=05%7C01%7Cxiaofan.jiang%40kcl.ac.uk%7C984c0a76e7944c8f9b7f08da8b555c97%7C8370cf1416f34c16b83c724071654356%7C0%7C0%7C637975496001456729%7CUnknown%7CTWFpbGZsb3d8eyJWIjoiMC4wLjAwMDAiLCJQIjoiV2luMzIiLCJBTiI6Ik1haWwiLCJXVCI6Mn0%3D%7C3000%7C%7C%7C&sdata=%2Bnv2NnYlDpqyXAXOQ6TSXIZQP0UYim%2BXmeTO1kllvQg%3D&reserved=0) **which is a pre-print of a paper that performs a GWAS on vascular tortuosity in the UK Biobank. Of note this work is still in pre-print only, however.**

*We had already cited the Zekavat et al paper in the Discussion, but have now added this citation to the Introduction and have expanded upon the statement about novelty. We are aware of this MedRxiv paper by Tomasoni et al, but had not cited this particular work because it is not peer reviewed, which we understand is against the editorial policy.*

**Page 6 lin 223: COL4A1 (next to COL4A2) is the gene thought to be causal of the medialian disorders FRAT and HANAC. I’m wondering why FRAT is not mentioned in this paper (HANAC is less well known but also thought to be caused by COL4A1 mutation). FRAT is a well known genetic cause of vascular tortuosity. It’s interesting that COL4A2, the neighboring gene, comes up – perhaps the authors can comment on this – are we wrong about COL4A1 being the cause of FRAT? Or is the authors’ signal actually tagging COL4A1? How are COL4A1 and COL4A2 related intracellularly?**

*We have added a reference to COL4A1 in the newly revised version of the manuscript (page 9, lines 369-372): “Interestingly, the COL4A2 gene is located next to the COL4A1 gene on chromosome 13q34. COL4A1 is known to harbour mutations that cause familial retinal arteriolar tortuosity.^4^ It is therefore unclear if COL4A1, COL4A2 or both can contribute to changes in the retinal vasculature.”*

**Page 7 line 247: Great that the authors replicate their work in this paper. However, the text should be more specific. They report a “generally linear relationship” between effect sizes – please report the specific correlation with confidence interval and p-value, which should be strong based on the supplementary figure.
Page 7 line 258: report p-value and confidence interval for correlations with other ancestries**

*We thank the reviewer for these helpful suggestions. Two additional plots showing the relationship between effect sizes were added as Supplementary Figure 3. That information has now been added to the main text (page 7, lines 277-278, i.e., “correlations between effect sizes observed in subjects of European and African (Pearson’s r = 0.33, 95% CI [0.12, 0.51], p =0.02) and South Asian ancestry (r = 0.46, 95% CI [0.27, 0.61], p <0.00001).”*

**Page 7 line 280: can the authors be more statistically specific – what is the exact p-value, how many SNPs/eQTLs were tested, what is the resulting Bonferroni cutoff.**

*The number of the SNP of each MR test is made more visible in the Supplementary Table 10, in the “num. instruments” column. Because the eQTLs are very correlated between tissues, we did not carry out Bonferroni correction. The p-values and FDR were those reported by the GTEx project, the latter correcting for multiple testing.*

**Page 8 line 293: The discussing of two-sample MR analysis to assess causality is really interesting. Yet, certain clinical observations make it hard to reconcile that retinal arteriolar tortuosity causes elevated diastolic blood pressure. A mechanical elevation in systemic blood pressure via microvasculature is more believable in the kidneys, for example, where there is a large enough tissue mass to actually affect systemic blood pressure. But the retinal arteries are massively smaller in quantity. Can the authors please provide more details as to how well powered they were to decide about causality direction? Could there be an unmeasured confounder here? For example, in the situation of RVO (2% prevalence) or impending RVO, we know that vascular tortuosity comes second, with elevated blood pressure being in the causal pathway. On longitudinal patient exam, the vessels are normal, then there is uncontrolled hypertension which leads to thickening of arteriolar walls and subsequent compression of the vein, resulting in venous tortuosity with associated RVO or impending RVO. To me, this seems like the opposite direct as is described by the authors.**

*We thank the reviewer for this observation and for highlighting the lack of clarity in our manuscript. One rationale for our study is that retinal vasculometry is just a biomarker of systemic vasculature and our MR results suggest that whatever processes that affect retinal vasculometry cause changes in systemic blood pressure and other traits. We have carefully tried to avoid giving the impression that retinal vasculature changes that can alter systemic parameters. We have now edited the manuscript to reinforce this message and avoid further misunderstanding.*

**References**

1.	Zheng J, Erzurumluoglu AM, Elsworth BL, Kemp JP, Howe L, Haycock PC, Hemani G, Tansey K, Laurin C, Pourcain BS, Warrington NM, Finucane HK, Price AL, Bulik-Sullivan BK, Anttila V, Paternoster L, Gaunt TR, Evans DM, Neale BM. LD Hub: a centralized database and web interface to perform LD score regression that maximizes the potential of summary level GWAS data for SNP heritability and genetic correlation analysis. *Bioinformatics.* 2017;33(2):272-279.

2.	Zekavat SM, Raghu VK, Trinder M, Ye Y, Koyama S, Honigberg MC, Yu Z, Pampana A, Urbut S, Haidermota S, O'Regan DP, Zhao H, Ellinor PT, Segre AV, Elze T, Wiggs JL, Martone J, Adelman RA, Zebardast N, Del Priore L, Wang JC, Natarajan P. Deep Learning of the Retina Enables Phenome- and Genome-Wide Analyses of the Microvasculature. *Circulation.* 2022;145(2):134-150.

3.	Welikala RA, Fraz MM, Foster PJ, Whincup PH, Rudnicka AR, Owen CG, Strachan DP, Barman SA, Consortium UBEV. Automated retinal image quality assessment on the UK Biobank dataset for epidemiological studies. *Computers in Biology and Medicine.* 2016;71:67-76.

4.	Zenteno JC, Crespi J, Buentello-Volante B, Buil JA, Bassaganyas F, Vela-Segarra JI, Diaz-Cascajosa J, Marieges MT. Next generation sequencing uncovers a missense mutation in COL4A1 as the cause of familial retinal arteriolar tortuosity. *Graefes Arch Clin Exp Ophthalmol.* 2014;252(11):1789-1794.
